# Decontamination treatments to eliminate problem biota from macroalgal tank cultures of *Osmundea pinnatifida*, *Palmaria palmata* and *Ulva lactuca*

**DOI:** 10.1007/s10811-016-0873-9

**Published:** 2016-07-01

**Authors:** Philip D. Kerrison, Hau Nhu Le, Gail C. Twigg, Duncan R. Smallman, Rory MacPhee, Fiona A. B. Houston, Adam D. Hughes

**Affiliations:** 1 SAMS, Scottish Marine Institute, Dunbeg, Argyll PA37 1QA UK; 2 Nha Trang Institute of Technology and Application (NITRA), Vietnam Academy of Science and Technology (VAST), 2 Hung Vuong Street, Nha Trang City, Vietnam; 3 SAMS, Scottish Marine Institute, Dunbeg, Argyll PA37 1QA UK; 4 Slate Islands Seaweed Ltd, 1B Easdale Island, Oban, Argyll PA34 4TB UK; 5 Celtic Sea Spice Co, 6 Long Craig Rigg, West Shore Road, Edinburgh, EH5 1QT UK

**Keywords:** Decontamination, Epibiont, Epiphyte, Hypochlorite, Iodide, Macroalgae

## Abstract

The effect of a range of chemical disinfectants at different concentration and exposure times was investigated on five macroalgal species and the marine gastropod *Littorina* spp. *Palmaria palmata*, *Osmundea pinnatifida* and *Ulva lactuca* are commercially valuable and are often cultivated in tanks for food or feed. *Ectocarpus siliculosus* and *Ulva intestinalis* are common epiphytes of *P. palmata* and *O. pinnatifida* cultures, whilst *Littorina* spp. are common herbivorous epibionts within *U. lactuca* culture tanks. These contaminants reduce the productivity and quality of the culture as a food. Differential tolerance to the treatments was seen between the algal species using pulse-amplitude modulation (PAM) chlorophyll *a* fluorescence, a few hours and a week following treatment. We identified treatments that selectively damaged the epiphyte but not the basiphyte species. *Ectocarpus siliculosus* had a significantly lower tolerance to 1 % sodium hypochlorite than *P. palmata*, and to 25 % methanol than *O. pinnatifida*, with a 1–5 min exposure appearing most suitable. *Ulva intestinalis* had a significantly lower tolerance than *P. palmata* and *O. pinnatifida* to many disinfectants: 0.1–1 % sodium hypochlorite for 10 min, 0.5 % potassium iodide for up to 10 min, and 0.25 % Kick-start (a commercial aquaculture disinfectant solution) for 1–5 min. No treatment was able to kill the gastropod snails without also damaging *U. lactuca*, although agitation in freshwater for an hr may cause them to detach from the basiphyte, with little to no photophysiological impact seen to *U. lactuca*. This experiment forms the basis for more extended commercial trials.

## Introduction

Many macroalgal species have high commercial value as food products (FAO 2012). Natural harvesting is unable to satisfy this demand and can cause ecological damage to coastal ecosystems (Hughes et al. 2013). This has driven the development of cultivation, making macroalgae the largest aquaculture product by volume in the world: 19 million tonnes, with an estimated value of US$5.7 billion (FAO 2012).

*Palmaria palmata* (L) Weber & Mohr and species of the genus *Ulva* spp. L have been investigated for the development of aquaculture (Ohno 2006; Hiraoka and Oka 2008; Edwards and Dring 2011). Tank cultivation of *Ulva* spp. has been attempted at various locations around the world, including Denmark, Florida, USA, Eilat, Israel, Zanzibar, Tanzania, South Africa and Australia (DeBusk et al. 1986; Msuya et al. 2006; Msuya and Neori 2010; Bruhn et al. 2011; Mata et al. 2016). Often these trials have been for its use as a biofilter to remove nutrients from sewage or aquaculture effluent, or as an animal fodder (Neori et al. 2003; Bolton et al. 2009; Msuya and Neori 2010; Al-Hafedh et al. 2015). *Palmaria palmata* is a popular seaweed for use in foods in North America and Europe (Martínez et al. 2006; Edwards and Dring 2011) and so its cultivation has also been attempted in onshore tanks (Morgan and Simpson 1981; Le Gall et al. 2004). *Osmundea pinnatifida* (Hudson) Stackhouse is another species with value as a food product, whose tank cultivation has recently been trialled (Rego et al. 2014).

Land-based tank cultivation as a reliable source of macroalgal biomass for food production will rely on the maintenance of positive vegetative growth and the quality of the stock. Reductions in growth and quality can occur due to contamination from fast growing epiphytes or grazing animals (Neish et al. 1977; Lüning and Pang 2003; Hwang et al. 2006). Diseases can also be responsible for degradation, such as two reported in tank-cultivated *Ulva* spp. (Colorni 1989; Del Campo et al. 2002).

The wild harvested seaweed used to stock cultivation tanks carries with it a diverse array of natural epiphytic flora and fauna. Without intervention, contaminating organisms can multiply, competing with the stock and/or causing deterioration in the quality, potentially leading to their collapse (Borowitzka 2007; Kerrison, unpublished results). The physical removal of these by washing and hand sorting substantially reduces their magnitude (Baweja et al. 2009); however, this is time-consuming and will never eliminate all epibionts. In addition, epibionts may be introduced later during seawater refreshment, by accidental cross-contamination or by wind-carried spores/eggs in the case of outdoor tanks. Therefore, efficient methods for the removal of such contaminants are essential. In some cases, manipulation of the culture conditions can prevent or minimise such contamination, such as high stocking densities, lower light and nutrient levels (Lüning and Pang 2003) or high pH and the release of alleochemicals caused by the target cultivation species itself (Björk et al. 2004; Gross 2010).

A chemical treatment may also be utilised to kill the contaminant. This relies on the existence of differential susceptibility, with a sensitive contaminant/s and a resilient cultivation species (Hoshaw and Rosowski 1973; Guillard 2007). Detergent, sodium hypochlorite (NaClO), reactive iodine, formaldehyde and organic solvents have been successfully utilised for the disinfection of macroalgal tissue to create axenic cultures (Shephard 1970; McCracken 1989; Baweja et al. 2009). Other compounds such as acids, peroxides, sodium hydroxide and commercial preparations, for example Virocid and Kick-start, are used in the aquaculture industry to disinfect equipment and prevent or treat disease (Togersen and Håstein 1995; Barge, personal communication), and so these may also be useful for decontamination in macroalgal cultures. As far as the authors are aware, the use of chemicals to remove contaminating epibionts from continuous macroalgal tank cultures has not been reported.

Chlorophyll *a* PAM fluorometry is a fast, sensitive and non-invasive technique which can probe the photosynthetic efficiency of organisms and has been commonly used for macroalgal studies (Kolber and Falkowski 1993; Enriquez and Borowitzka 2010). It is now used extensively to assess stress-dependent changes in photosynthesis of higher plants, micro- and macroalgae and cyanobacteria (Schreiber et al. 1986; Flameling and Kromkamp 1998; Figueroa et al. 2006). A relative measurement of algal photosynthetic health is obtained through measurement of the operating photosynthetic efficiency of photosystem II (F_q_′/F_m_′). Higher values indicate greater photosynthetic electron flow towards carbon fixation, while very low values indicate poor health or death (Maxwell and Johnson 2000; Cosgrove and Borowitzka 2010; Kerrison et al. 2016). This makes fluorometry a useful method to monitor the condition of the algal cell following a chemical treatment which may disrupt the delicate balance of cellular processes necessary for photosynthesis (Falkowski and Raven 2007). Such measurements have been used previously to assess the suitability of decontamination treatments on *Sargassum* spp. (Hwang et al. 2006; Pang et al. 2007; Kerrison et al. 2016).

The aim of this study is to identify treatments which could be utilised for the removal of specific biota from cultures of three commercially important macroalgae. *Ectocarpus siliculosus* (Dillwyn) Lyngbye and *Ulva intestinalis* L are fast growing epiphytes of *P. palmata* and *O. pinnatifida* in tank cultures (personal observation). The filamentous alga *E. siliculosus* can grow rapidly on the thalli of other macroalgae when supplied with ample light, temperature, nutrients and the absence of grazers (personal observation). *Ulva intestinalis* is a fast growing ephemeral algae, which can grow well in macroalgal culture where light and nutrients are plentiful, either detached or as an epibiont. It grows as thin tubular filaments composed of two-cell layers, which can reach 50 cm in length, smothering and outcompeting other macroalgae. *Littorina* spp. gastropods are small (<10 mm) highly active grazers within cultures of *Ulva lactuca* which can occur at very high densities, making hand removal unfeasible (personal observation). To accomplish the aim, we will test the physiological impact of many commercially available disinfectants to each of these species, over a range of concentration and exposure times. In the macroalgae, fluorometry will be used to monitor their photosynthetic health, and survival will be monitored in the gastropod.

## Materials and methods

The tolerance of the cultivated and most common epibiont species were examined in three combinations: (1) sections of *P. palmata* (2–3 cm^2^) with epiphytic *E. siliculosus*, (2) a sprig (2–3 cm) of *O. pinnatifida* with *U. intestinalis* (2–3 strands), and (3) a section of *U. lactuca* (2–3 cm^2^) and two to three grazing snails (4–6 mm shell diameter), a mixture of *Littorina litorea* Linnaeus, and *Littorina obtusata* Linnaeus. Visibly healthy units from each species (be it 2–3 cm^2^ section, sprigs or individuals) were selected from outdoor tank culture at Otter Ferry Seafish Ltd (Argyll, UK). These were exposed to a range of disinfectants over different concentrations and for either 1, 5, 10, 30 or 60 min (Table 1).

**Table 1:** Characteristics of seventeen chemical agents with potential for the decontamination of macroalgal cultures

Chemical decontaminant	Action	Exposure time			Supplier
		1/5 min (%)	10 min (%)	30/60 min (%)	
Distilled water	Osmotic shock to cells	100	100	100	n/a
Detergent_FW_ Decon®90	Membrane disruption and cellular lysis		1, 2	1, 2, 5	Decon Laboratories Ltd, Sussex, UK
Sodium hypochlorite_SW_	Antimicrobial. Oxidative damage by generation of hydroxyl radicals	0.1, 1, 2, 5	0.1, 1, 2		Sigma Aldrich Co Ltd, UK (133440)
Ethanol_FW_	Antimicrobial, dehydration, cell lysis and protein coagulation	25, 50, 75	25, 50, 75		Sigma Aldrich Co Ltd, UK (02883)
Methanol_FW_	Antimicrobial, dehydration, cell lysis and protein coagulation	25, 50, 75	25, 50, 75		Sigma Aldrich Co Ltd, UK (34860)
Hexane	dehydration, protein coagulation		100	100	Sigma Aldrich Co Ltd, UK (296090)
Isopropanol_FW_	Antimicrobial, dehydration, cell lysis and protein coagulation	25, 50, 75	25, 50, 75		Sigma Aldrich Co Ltd, UK (278475)
Potassium iodide_SW_	Oxidative damage by generation of hydroxyl radicals.	0.5, 1, 2	0.5, 1, 2		Sigma Aldrich Co Ltd, UK (207772)
Lugol’s iodine_SW_	Oxidative damage by generation of hydroxyl radicals	0.1, 0.25, 0.5	0.25, 0.5, 1		Recipe in Sherr and Sherr (1993)
Hydrogen peroxide_SW_	Mild antiseptic. Oxidative damage.	0.5, 1, 2	0.5, 1, 2		Sigma Aldrich Co Ltd, UK (216763)
Acetic acid_SW_	Acid reduction of cellular components	0.1, 0.25, 0.5	0.5, 1, 2		Sigma Aldrich Co Ltd, UK (320099)
Paracetic acid_SW_	Acid reduction of cellular components	0.1, 0.25, 0.5	0.5, 1, 2		Sigma Aldrich Co Ltd, UK (320099 and 216763)
Virocid_SW_ (alkyldimethylbenzyl ammonium chloride, didecyldimethyl ammonium chloride, glutaraldehyde and isopropanol)	Biocide and cationic surfactant. Antimicrobial and antiviral. Toxic and carcinogenic. Antimicrobial, dehydration, cell lysis and protein coagulation	0.1, 0.25, 0.5	0.25, 0.5, 1, 2		CID Lines N.V., BE
Kickstart_SW_ (H_2_O_2_, acetic acid and peracetic acid)	Mild antiseptic. Oxidative damage. Acid reduction of cellular components	0.25, 0.5, 1	0.25, 0.5, 1		CID Lines N.V., BE
pH 2.5 (sodium hydroxide)_SW_	Basic oxidation of cellular components		100	100	Sigma Aldrich Co Ltd, UK (S5881)
pH 10.5 (hydrochloric acid)_SW_	Acid reduction of cellular components		100	100	Sigma Aldrich Co Ltd, UK (320331)
dichloroisocyanurate dehydrate_SW_ Clearwater™	Reactive chlorine. Oxidative damage		0.05, 0.02, 0.1	0.1, 0.5, 1	Complete Pool Controls Ltd, Bishops Cleeve, UK

The activity of the agent responsible for this potential is given, as is the supplier used in this study and the concentrations (%) examined under each exposure time. The dilutant is either distilled water (_FW_) or Tyndallised seawater (_SW_). All of these chemical agents have the potential to disrupt algal photosynthesis. Concentration ranges tested were based on the results of previous experimentation

The procedure followed that of Kerrison et al. (2016). Each species-chemical-concentration-exposure time combination (*n* = 1 for each) was submerged in polystyrene Petri dishes containing 20 mL volumes of a disinfectant solution (see Table 1). After the allotted exposure time, these were transferred using forceps to 150 mL of UV-sterilised seawater and agitated for a few seconds to rinse off any of the remaining disinfectant. After 5–10 min, these were transferred into fresh dishes containing 30 mL of F/2 medium without silicate in Tyndallized seawater (Kawachi and Noël 2005). The dishes were then incubated at 8.5 °C and 10–15 μmol photons · m^−2^ · s^−1^ with a 12:12-h light/dark cycle. Each species-chemical concentration-exposure time combination was examined only once (*n* = 1 for each). The control for each species underwent the same protocol except they were only exposed to seawater. These were replicated (*n* = 5) to ensure that each treatment were compared against a precise base value.

The trial was first performed with a 10-min exposure to distilled water; 1–5 % detergent; 0.1–2 % NaClO; 25–75 % ethanol, methanol and isopropanol; 100 % hexane; 0.5–2 % saturated KI solution, 0.25–1 % Lugol’s iodine; 0.5–2 % H_2_O_2_, acetic acid and paracetic acid; 0.25–2 % Virocid and Kick-start; pH 2.5 or 10.5 (adjusted with hydrochloric acid or sodium hydroxide); and 0.01–0.1 % dichloroisocyanurate dehydrate. The organic solvents (ethanol, methanol and isopropanol) and detergent were diluted in distilled water, while all others were diluted in 10 μm filtered, UV-sterilised seawater. The concentrations chosen were based on the effective ranges shown by other studies; reference to these is made in the discussion. The test conditions, purchasing source and disinfectant activity of each chemical are listed in Table 1.

The operating efficiency of photosystem II (F_q_′/F_m_′) was measured in triplicate on each treated seaweed using an Aquapen-P fluorometer (Photon Systems Instruments, Brno, Czech Republic) 1–2 h following treatment and again after 7 days of incubation. The relative operating efficiency (rF_q_′/F_m_′) was then calculated as a % of mean F_q_′/F_m_′ measured in the replicate controls. The effect of the disinfectant was then coded by effect: supra-optimal (>130 %), minimal-none (70–130 %), moderate (30–<70 %), severe (5–<30 %) or lethal (<5 %). These ranges are based on those identified by Kerrison et al. (2016).

Post-treatment recovery after 1 week (ΔrF_q_′/F_m_′) was calculated as the change in F_q_′/F_m_′, between the two time points. These were coded as follows: excellent (>50 %), moderate (10–50 %) or no recovery (−10 to <10 %).

Minitab v.15 (Minitab Inc.) was used for all statistical analyses. Three-way analysis of variance (3wAN) was used to test for significant differences in the rF_q_′/F_m_′ response to each disinfectant, with species, concentration and exposure time. Two-way analysis of variance (2wAN) was used where concentration did not change, e.g. A, 100 % distilled water.

Due to the lack of replication of each species-chemical concentration-exposure time combination (*n* = 1), it was not possible to directly test the data for the homogeneity of variance and normality. In this situation, it is recommended to test similar data that can be validated (Sokal and Rohlf 1995; Dytham 2003). The replicated controls (*n* = 5 for each species) used in this experiment satisfied both the Anderson-Darling test for normality (Anderson and Darling 1952) and the Levene’s test for homoscedasticity (Levene 1960). In addition, the same result was found in a separate replicated dataset examining the effects of NaClO and potassium iodide (KI) on *Sargassum muticum* (Kerrison et al. 2016).

Combinations of disinfectant, concentration and exposure time were identified where the cultivated basiphyte (*P. palmata*/*O. pinnatifida*) was minimally or not affected (rF_q_′/F_m_′, 70–130 %) whilst the epiphyte (*E. siliculosus*/*U. intestinalis*) was severely or lethally affected (rF_q_′/F_m_′, <30 %). For each specific treatment concentration, 3wAN was then used to test for significance in the rF_q_′/F_m_′ response between the species pairs, across exposure time and at both time point (1–2 h and 7 days).

## Results

### *Littorina* spp. survival

No significant effect was seen in gastropod survival between the different treatments (*p* > 0.05), with only a few fatalities evident, mainly in the isopropanol and Virocid treatments. This meant that no treatment could be suggested for the removal of gastropods from *U. lactuca* culture.

### Distilled water

This treatment was quite benign to all species examined, with no significant difference shown (*p* > 0.05). A significant effect was seen between the 10-, 30- and 60-min exposures (2wAN: *F*_2,4,8,14_ = 13.6, *p* < 0.005). rF_q_′/F_m_′ was 13–54 % higher than the control after 10 min in all species, but after 60 min, all were <100 %, with a moderate effect seen in *E. siliculosus*, *P. palmata* and *O. pinnatifida*. After a wk, all had recovered and little-no effect was observed (rF_q_′/F_m_′ within 70–130 %), this indicates that distilled water was an ineffectual treatment.

### Detergent

The exposure time (3wAN: *F*_2,4,1,22_ = 42.2, *p* < 0.0001) and concentration (3wAN: *F*_2,4,1,22_ = 42.2, *p* < 0.0001) of detergent were both significant. A 10-min exposure to 0.5–2 %, lead to supra-optimal rF_q_′/F_m_′ in some species, but generally appeared benign (Table 2). After 30 min, a moderate, severe or lethal effect was observed, particularly at the highest concentration. After 60 min, this was exacerbated with death in half of the samples, particularly at 2–5 %. *Ulva lactuca* appeared highly tolerant, coping with 60 min in 5 % detergent with only a moderate effect, which was lethal in all other species. However, no species-specific effect was seen between treatments (*p* > 0.05).

**Table 2:**
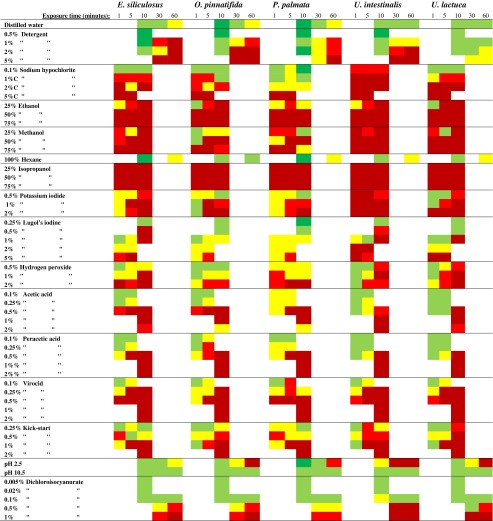
Coded operating photochemical efficiency response (F_q_′/F_m_′) of five macroalgal species exposed to a range of chemical disinfections at different concentrations for between 1 and 60 min

These are *E. siliculosus*, *O. pinnatifida*, *P. palmata*, *U. intestinalis* and *U. lactuca*. The % relative to the control (rF_q_′/F_m_′) post-treatment were colour coded by effect: >130 % supra-optimal , 70–130 % minimal-none , 30–<70 % moderate , 5–<30 % severe  and <5 % lethal

### Sodium hypochlorite (NaClO)

The response to NaClO was significantly different between species (3wAN: *F*_2,4,3,50_ = 2.0, *p* < 0.001) and with concentration (3wAN: *F*_2,4,3,50_ = 6.2, *p* < 0.0001), but not exposure time (*p* > 0.05). *Ulva intestinalis* was always severely or lethally affected, while the highest tolerance was found in *P. palmata*. A 1 min in 5 % was lethal in all species.

### Ethanol

The exposure time (3wAN: *F*_2,4,2,36_ = 5.6, *p* < 0.01) and concentration (3wAN: *F*_2,4,3,50_ = 15.2, *p* < 0.0001) had significant effects, but no species effect was seen (*p* > 0.05). Fifty to 75 % always had a lethal effect at 1–10 min. At 25 %, severe to lethal effects were seen after 5–10 min, with some moderate effects seen after 1 min.

### Methanol

Methanol usually had a severe impact. Only the concentration of methanol had a significant effect (3wAN: *F*_2,4,3,50_ = 7.2, *p* < 0.005) with mainly lethal impacts seen at 50–75 %.

### Hexane

No significant effect was observed due to time or species (*p* > 0.05).

### Isopropanol

Isopropanol was lethal at all concentration and exposure times.

### Potassium iodide (KI)

All factors were significant in the KI treatment. *Ulva intestinalis* was most severely affected, while *O. pinnatifida* and *U. lactuca* were most tolerant (3wAN: *F*_2,4,2,36_ = 4.1, *p* < 0.01). Both longer exposure time (5–10 min) and higher concentration (1–2 %) lead to severe-lethal effects (3wAN: *F*_2,4,2,36_ = 6.2–10.6, *p* < 0.005–0.0001).

### Lugol’s iodine

No species or exposure time effect was observed due to Lugol’s iodine (*p* > 0.05). Concentration was significant (3wAN: *F*_1,4,2,22_ = 24.8, *p* < 0.0001), with the higher % concentration leading to stronger negative effect.

### Hydrogen peroxide (H_2_O_2_)

A significant species effect was observed due to H_2_O_2_ (3wAN: *F*_2,4,2,36_ = 3.9, *p* < 0.01), with higher sensitivity seen in *P. palmata* and *E. siliculosus* and lowest effect in *O. pinnatifida.* Both higher concentration (1–2 %) and exposure time (10 min) lead to the greatest negative effect (3wAN: *F*_2,4,2,36_ = 14.7–20.8, *p* < 0.0001).

### Acetic acid

A highly significant effect was observed due to species (3wAN: *F*_1,4,2,22_ = 9.9, *p* < 0.0001), with little to no effect on *U. intestinalis* and the largest effect seen in *P. palmata*. Another significant effect was seen due to concentration (3wAN: *F*_1,4,2,22_ = 22.7, *p* < 0.0001), with higher concentrations leading to the strongest effect.

### Peracetic acid

A species-specific difference was observed (3wAN: *F*_1,4,2,22_ = 6.3, *p* < 0.005) with *P. palmata* and *O. pinnatifida* being more sensitive. Both exposure time and concentration were also significant (3wAN: *F*_1,4,2,22_ = 12.6–21.4, *p* < 0.0001), with highest exposure time (5–10 min) and concentration (0.5 %) leading to moderate-severe effects.

### Virocid

No significant difference was observed between species (*p* > 0.05). Both exposure time and concentration were significantly affected (3wAN: *F*_2,4,2,36_ = 52–59, *p* < 0.0001), with higher exposure time (5–10 min) and concentration (0.5–2 %) leading to severe effects.

### Kick-start

No significant difference was observed between species (*p* > 0.05). Both exposure time and concentration were significant (*F*_2,4,2,36_ = 7.9–11.7, *p* < 0.001–0.0001), with higher exposure time (5–10 min) and concentration (1 %) leading to lethal effects.

### pH 2.5 or 10.5

No significant effect was seen regarding species or exposure time (*p* > 0.05).

### Dichloroisocyanurate

No significant difference was seen with the exposure time or species (*p* > 0.05). There was a significant difference due to the concentration (*F*_1,4,2,22_ = 76.5, *p* < 0.0001). Minimal to no effect was seen at 0.005–0.1 % while only severe to lethal effects were seen at 1 %.

### Comparison of disinfectant effect: *P. palmata* versus *E. siliculosus* and *U. intestinalis*

Seven treatments lead to minimal to no effect in *P. palmata* and a severe-lethal effect in *E. siliculosus* and/or *U. intestinalis* and so are potentially suitable for the selective removal of these problem species.

**Fig. 1:**
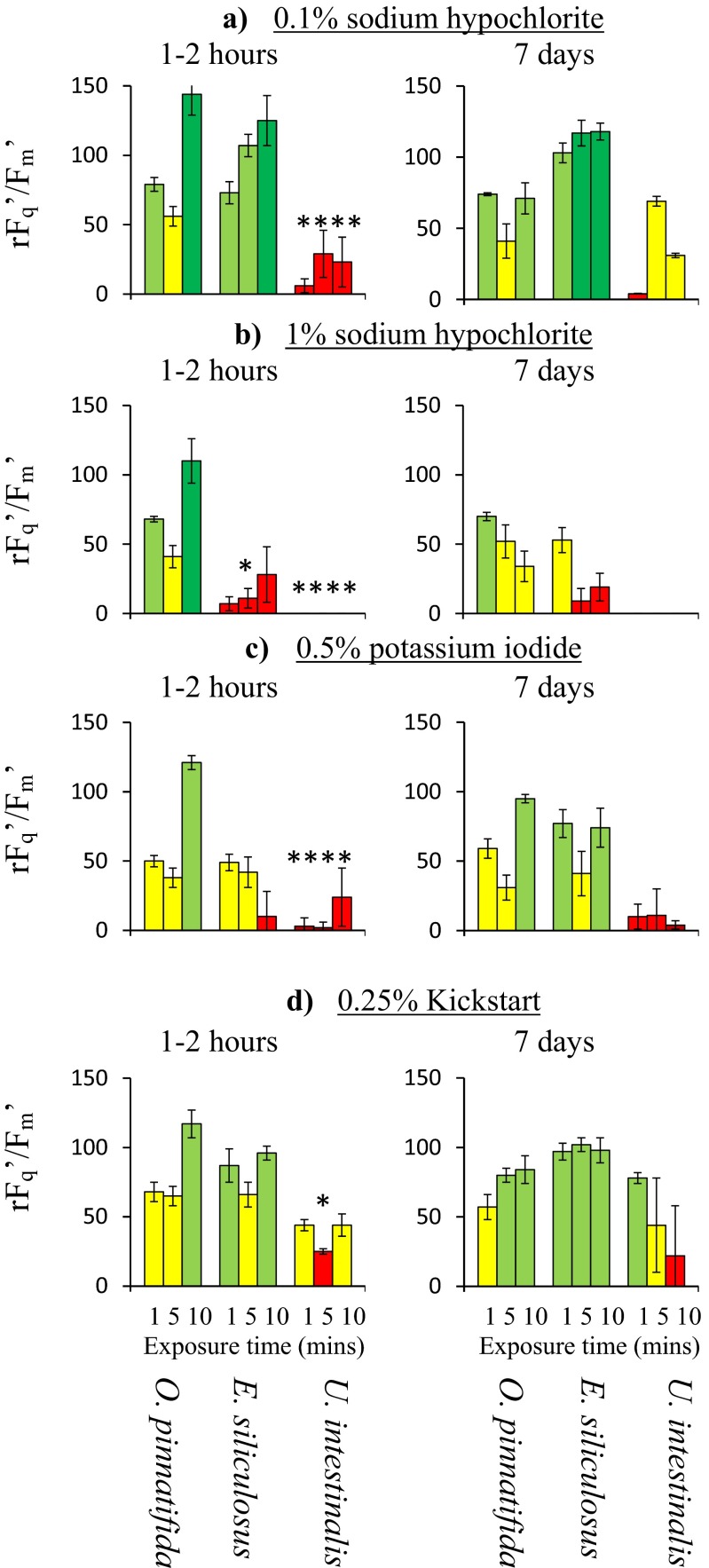
Relative change in the operating efficiency of photosystem II (rF_q_′/F_m_′) of *O. pinnatifida* and two epiphytic species: *E. siliculosus* and *U. intestinalis* to different chemical treatments over three exposure times (1–10 min) and at two measurement points (after 1–2 h or 7 days, shown in separate graphs). Significant reductions in either epiphytic species compared to *O. pinnatifida* are denoted by *p < 0.05, **p < 0.01, ***p < 0.005 and ****p < 0.001. Shown is mean ± standard deviation (pseudo-replicated measurements on a single individual)

The only treatment that had a significantly greater effect on *E. siliculosus*, than *P. palmata* was 1 % NaClO (Fig 1; 3wAN: *F*_1,2,1,7_ = 8.8, *p* < 0.05), with between 1 and 5 min looking highly suitable. NaClO also had a significantly greater effect on *U. intestinalis* than *P. palmata* at two concentrations: 0.1 % (3wAN: *F*_1,2,1,7_ = 7.3, *p* < 0.05) and 1 % (3wAN: *F*_1,2,1,7_ = 27.3, *p* < 0.001). The most suitable exposure time appeared to be either 1–10 or 1–5 min, respectively. KI at 0.5 % for 1 min was also suitable (3wAN: *F*_1,2,1,7_ = 23.3, *p* < 0.005) as was 0.25 % Kick-start for 5 min (3wAN: *F*_1,2,1,7_ = 6.8, *p* < 0.05).

Both 0.5 % Kick-start and pH 2.5 did not give any significant effect (*p* > 0.05). Lugol’s (0.5 %) for 10 min appeared potentially effective against both *E. siliculosus* and *U. intestinalis*, but was not significant (*p* > 0.05).

### Comparison of disinfectant effect: *O. pinnatifida* versus *E. siliculosus* and *U. intestinalis*

Seven treatments were also highlighted as potential disinfectants for *O. pinnatifida* as they lead to minimal to no effect in this species but severe-lethal effect in either *E. siliculosus* and/or *U. intestinalis*.

**Fig. 2:**
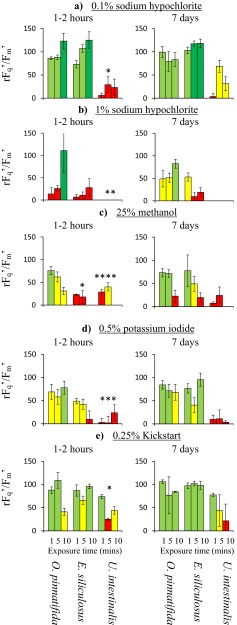
Relative change in the operating efficiency of photosystem II (rF_q_′/F_m_′) of *O. pinnatifida* and two epiphytic species: *E. siliculosus* and *U. intestinalis* to different chemical treatments over three exposure times (1–10 min) and at two measurement points (after 1–2 h or 7 days, shown in separate graphs). Significant reductions in either epiphytic species compared to *O. pinnatifida* are denoted by *p < 0.05, **p < 0.01, ***p < 0.005 and ****p < 0.001. Shown is mean ± standard deviation (pseudo-replicated measurements on a single individual)

Only 25 % methanol had a significantly greater effect on *E. siliculosus* than *O. pinnatifida* (Fig. 2; 3wAN: *F*_1,2,1,7_ = 8.7, *p* < 0.05), with a 1-min exposure appearing the most suitable. However, there was significant recovery of rF_q_′/F_m_′ (3wAN: *F*_1,2,1,7_ = 12.7, *p* < 0.01), especially in *O. pinnatifida* between the two measurement points; 1 % NaClO, 25 % ethanol, 50 % methanol, 0.5 % Lugol’s iodine, 1 % H_2_O_2_ and 0.25 % Kick-start did not show significance (*p* > 0.05).

*Ulva intestinalis* was not significantly more sensitive than *O. pinnatifida* to either 0.5 % Lugol’s iodine or 1 % H_2_O_2_ (*p* > 0.05). It was significantly more sensitive to the other five highlighted treatments:

1. 0.1 % NaClO (3wAN: *F*_1,2,1,7_ = 7.0, *p* < 0.05), with exposure of up to 10 min appearing suitable.
2. 1 % NaClO (3wAN: *F*_1,2,1,7_ = 14.9, *p* < 0.01), with 10 min appearing suitable.
3. 25 % methanol (3wAN: *F*_1,2,1,7_ = 42.6, *p* < 0.0001) with a 1–5-min exposure appearing suitable.
4. 0.5 % KI (3wAN: *F*_1,2,1,7_ = 17.0, *p* < 0.0005) with 1 min appearing suitable.
5. 0.25 % Kick-start (3wAN: *F*_1,2,1,7_ = 10.2, *p* < 0.05) with a 5-min exposure appearing most suitable.

## Discussion

Large macroalgal cultures can suffer from contamination and overgrowth by epibionts introduced during tank stocking or from the surrounding environment (Lüning and Pang 2003). This is comparable to the situation regarding microalgal pond cultivation where contamination by various biota is a continual problem (Richmond 2004). Such contaminants can reduce growth of the cultured species, reduce the quality/quality of the biomass produced or cause the culture to collapse entirely. Methods to prevent or eliminate epiphytic contamination in large macroalgal tanks are necessary so that tank productivity and product quality are maximised. Chemical treatments have been successfully utilised to reduce or remove contaminants (Guillard 2007); however, treatment success is dependent on careful control of the dosing so as to inhibit the growth of, or kill the contaminant/s but not unduly damage the cultured species (Hoshaw and Rosowski 1973). Therefore, these treatments must be tailored to the specific cultured algae and contaminant (Hoshaw and Rosowski 1973). For instance in the case of microalgae, 4–10 ppm of active chlorine can be used to remove protozoan grazer from cultures of *Nannochloropsis* spp. (Richmond 2004), and dosing of 2,4-dinitro-6-cyclohexyl phenylacetate or pentachlorophenylacetate can remove many contaminants from *Chlorella ellpsoidea* (Tamiya 1955).

Previous studies on the chemical treatment of macroalgal tissue have tended to focus on the disinfection of thallus material to obtain unialgal cultures within a particular species or species group, often testing only one or few different chemicals at high concentration and/or exposure times (i.e. Druehl and Hsiao 1969; Yan 1984; Aguirre-Lipperheide and Evans 1993). These aggressive treatments can severely impact the physiology of macroalgae as they do not possess a protective cuticle, and so are highly susceptible to chemical damage (Fries 1980; Baweja et al. 2009; Fernandes et al. 2011). However, since the objective of such studies has been the isolation of axenic tissue, this physiological impact is acceptable, as long as the macroalga recovers successfully, allowing continued cultivation. In the case of large, land-based tank cultures, continuous tank productivity is preferred to maximise profitability. So, it is necessary that the dosing of any chemical treatments is carefully tuned so as to eliminate epibionts, but with little physiological damage to the target species.

There is very little information available on which to compare the tolerance of different macroalgal species, such as co-existing basiphyte and epiphytes. The exception to this is Kientz et al. (2012) who tested various disinfectants, concentrations and immersion times on five macroalgae including *P. palmata* and *U. lactuca*. However, their aim was the removal of microbial epibionts, and so the treatment effectiveness at removing macroscopic epibionts was not assessed and neither was the physiological condition of the basiphyte post-treatment. The authors are not aware of any previous study or reports which have attempted to systematically determine the effectiveness of chemical treatments for the removal of macroscopic epibionts from the species *P. palmata*, *O. pinnatifida* and *U. lactuca*.

This study has used the photophysiological response of five macroalgae to identify suitable chemical treatments. A wide range of conditions were examined—17 chemicals each at up to five exposure concentrations, over three exposure times (1, 5, 10 or 10, 30, 60 min) in six species. Because an expansive study was chosen, each combination was not replicated (*n* = 1), other than the controls (*n* = 5). Therefore, further testing is necessary to confirm the suitability of the highlighted candidate treatment before being utilised at a commercial scale.

In a separate study, Kerrison et al. (2016) used the same method to collect data on the chemical tolerance of the phaeophyte *S. muticum*. By contrasting their results with the present study, they determined that *S. muticum* had a high tolerance to reactive chlorine and iodine compounds, making these potentially suitable decontaminants for a *S. muticum* hatchery. Further testing then confirmed this and found that a combined treatment of 0.5 % KI and 0.38 % NaClO for 3 min was effective at removing protozoan grazers from both adult and juvenile thalli, with minimal impact on photophysiology or juvenile growth. This demonstrates how the present dataset can be utilised as a basis for further study, grounded on the differential tolerance seen in the macroalgal species tested.

The present study successfully identified significant differential tolerance between the epiphyte and basiphyte combinations. Only two treatments showed promise for the removal of *E. siliculosus*: 1 % NaClO for 1–10 min in cultures of *P. palmata*, and 25 % methanol for 1–5 min in *O. pinnatifida*. In general, *E. siliculosus* had a similar photophysiological response to *P. palmata* and *O. pinnatifida*, making it difficult to selectively damage. This was surprising, since *E. siliculosus* is composed of filaments, with a high surface area, and so it was reasoned it would be more susceptible to chemical attack. The result supports the reported ‘physiological toughness’ of this species, as illustrated by its salinity and copper tolerance (Charrier et al. 2008).

This contrasts with the results seen in *U. intestinalis*; although this species has high tolerance to desiccation, light and temperature (Vadas et al. 1977; McAllen 1999), it was very sensitive to many chemical treatments and so multiple candidate treatments were identified for its removal from *P. palmata* and *O. pinnatifida*. These include 0.1–1 % NaClO for 1–10 min, 0.5 % KI for 1–10 min (less suitable for *O. pinnatifida*) and 0.25 % Kick-start for 5–10 min. Of these, the most promising candidate treatments for both species would appear to be the following: a 10 min in 0.1 % NaClO, a 1-min exposure to 1 % NaClO, a 10-min exposure to 0.5 % KI or a 10-min exposure to 0.25 % Kick-start.

### Previous studies involving NaClO, methanol, KI and other iodide compounds and Kick-start

NaClO is commonly available and has often been effectively utilised within protocols aimed at producing unialgal or axenic macroalgal cultures (Baweja et al. 2009). Usually, this involves low concentrations, i.e. 1 %, for up to 30 min (Druehl and Hsiao 1969; Lee 1985), although 5 % or saturated solutions have been used for up to 5 min (Hsiao and Druehl 1971; Fries 1977). Despite its common usage, it oxidant activity can easily damage tissues leading to softening and/or pigment loss (Baweja et al. 2009; Fernandes et al. 2011). This study has shown that the damage takes some time to repair, with little recovery seen a wk afterward (Figs. 1 and 2). This characteristic may make it a very suitable compound for disinfection, as damage to a contaminating epiphyte will persist, repressing the regrowth of any surviving tissue for some time after the treatment. It also means that careful dosing is essential, as any damage to the cultured species will also persist, reducing its future growth. This was seen to occur in juvenile *S. muticum* 20 days following treatment with 0.75 % NaClO for 3 min (Kerrison et al. 2016).

The present study has identified the solvent methanol is a candidate for the removal of *U. intestinalis* epiphytes from *O. pinnatifida*. Having said this, *O. pinnatifida* was moderately affected by a 5–10-min exposure (Fig. 2); this may make methanol too aggressive for use unless the concentration or exposure time is reduced further (<25 % and/or <1 min), although this may compromise its effectiveness. Methanol has only been tested in one previous disinfection study, where concentrations up to 80 % were found to not be effective at removing microscopic epibiota from a number of macroalgae (Kientz et al. 2012).

### Potassium iodide (KI) and other reactive iodine compounds

A 10-min exposure to 0.5 % KI was found to be effective for the removal of *U. intestinalis*. KI has been used successfully before in the disinfection of kelp (Yan 1984); however, a slightly high concentration of 1.5 % for 10 s was found to be lethal to *Ecklonia radiata* (Lawlor et al. 1991). In addition, KI can lead to iodine staining of the tissue (Kientz et al. 2012), which may affect the suitability and value of the treated tissue for food. Iodinated polyvinyl pyrrolidine, also known as Betaine, has also been frequently used in sterilisation protocols, often as a 0.5–1 % solution (Gibor et al. 1981; Lee 1985; Aguirre-Lipperheide and Evans 1993), while Lugol’s iodine can be used to remove diatoms from *Saccharina latissima* sporangia, 0.2 % for 2 min (Rød 2012). Our result from a 10-min exposure to 0.5 % Lugol’s iodine suggests that it may be useful for the removal of either *E. siliculosus* or *U. intestinalis*; however, this was not backed up by significant result, suggesting that KI is the more favourable iodine form to pursue.

The commercial disinfectant Kick-start is a mixture of H_2_O_2_, acetic and peracetic acid, recommended for the disinfection of aquaculture equipment and workspaces. Whilst none of the individual components alone was found to be suitable for epiphyte removal, when combined they were an effective treatment against *U. intestinalis*. This is the first report of their use on macroalgae.

### Lethal exposures in all macroalgae

A number of the disinfectant-exposure time-concentration combinations severely or lethally impacted all macroalgal species under investigation. This makes them highly unsuitable for the removal of specific epiphytes, as exposure was an effective algicide, causing damage both to all macroalgae tested, epiphyte and basiphyte. Lethal effects were observed after 1 min in 5 % NaClO, 50–75 % ethanol and 25–75 % isopropanol and 5 min in 0.5–2 % Virocid. Lethal effects were also usually observed after 1 min in 50–75 % methanol, 5 min in 1–2 % KI, 2 % H_2_O_2_, 1–2 % Kick-start, and 10 min in 0.5–2 % acetic acid, peracetic acid and dichloroisocyanurate. The algicidal effect of these components is useful information for those wishing to prevent the survival of a macroalgae culture, such as if working with an invasive species. In the fish aquaculture industry, a number of these components are already utilised effectively for the disinfection of tanks or workspaces (A. Barge, personal communication).

### Optimal exposure time

Other studies have utilised some of these algicidal compounds within disinfection protocols, with <1 min exposure time, allowing the survival of the macroalgae tissue. For instance, Lawlor et al. (1991) dipped *E. radiata* into 70 % ethanol for 30 s, Kawashima and Tokuda (1990) dipped into 70 % ethanol for 30–60 s and Kientz et al. (2012) recommend 30–60 s in 40–50 % ethanol and 1 % NaClO. Such short exposure times could be an effective treatments against the epiphytes reported. However, the motivation for the present study has been to develop protocols for the removal of epiphyte species from tank cultures containing large quantities of basiphyte biomass (tens kg). This makes short exposures unsuitable for two reasons. Firstly, the protocol requires a wide safety margin between effective removal of the epiphyte and damage to the basiphyte; for example, if a 30-s exposure is able to kill the epiphytes, but 60 s kills the basiphyte completely, an accidental overexposure could be catastrophic to the stock. Secondly, it is doubtful that such fine control on the exposure time, on the scale of minutes, is possible when exposing tens kg simultaneously.

On the other hand, very long exposures of an hr or more are also not ideal. The treatment should be completed relatively quickly, returning the macroalgae to optimal growth conditions, minimising the manpower required and allowing treatments of multiple tanks within a relatively short timeframe. Incidentally, none of the longer exposure treatments (30–60 min) were effective, suggesting that acute exposure is preferable. For these reasons, 5–10 min is considered the most favourable for the removal of epiphytes from large macroalgal cultures.

### Non-effective treatments

No severe effects were induced by some treatments: distilled water, hexane and pH 10.5. A number of the other chemicals also did not show any severe effects, when tested at low concentrations: 0.5 % detergent, 0.25 % Lugol’s, 0.1–0.25 % acetic acid, 0.1 % peracetic acid and 0.01 % dichloroisocyanurate. Whilst these were therefore not useful for the removal of the epiphytic species *E. siliculosus* or *U. intestinalis*, other uninvestigated organisms (i.e. other macroalgae, protozoans, fungi, bacteria, etc.) may be very sensitive to these treatments. Consequently, there may still be a benefit to the use of this treatments, i.e. fresh water treatment to kill organisms sensitive to osmotic shock (Kawai et al. 2007), since they do not provoke a negative effect in the physiology of the examined macroalgae. A full examination of this possibility is beyond the scope of the present study.

### *Littorina* spp. removal

No significant effect on survival was seen in the gastropod *Littorina* spp. and so it was not possible to suggest any candidate treatments for removal of this epibiont. In many cases, it was observed that when they were introduced to the potentially hazardous chemical environment of the treatment, the snails retracted into their shell, closing off the operculum (data not shown). This allowed them to insulate themselves from the treatment and so survive treatments such as 10 min in 75 % isopropanol, when exposed algal tissue was killed within a min. By closing the operculum, they can no longer use their pedal foot to grip firmly onto seaweed or other substratum. This may mean that to remove snails all that is needed, is a treatment that causes such retraction, combined with some agitation for them to become dislodged. Soaking in freshwater may therefore be an effective treatment.

Marine gastropods are osmoconformers which cannot regulate the osmotic strength of their haemolymph (Avens and Sleigh 1965). Upper littoral species have lower epithelial permeability giving them greater tolerance to osmotic stress and thus allowing them to remain active in low salinity (Avens and Sleigh 1965; Rumsey 1973). In a study by McMahon (2003), 16 out of 17 intertidal gastropod species examined responded with >50 % substratum detachment in freshwater, with most showing 50 % detachment at ~10–13 psu. This suggests that fresh water, which was quite benign to all the macroalgae examined in this study, combined with agitation may be all that is required to remove such snails from seaweed culture. *Ulva lactuca* is especially tolerant to such fresh water exposure and so a 1 h soak with regular agitation may be a very effective method for gastropod removal from this species.

## Conclusion

This study has shown that differential tolerance to chemical treatments exists within five macroalgal species. These significant differences have allowed us to identify treatments which selectively damage or inhibit the epiphytic species *E. siliculosus* and *U. intestinalis* while allowing cultures of *O. pinnatifida* and *P. palmata* to be left healthy. Specific treatments (chemical, concentration and exposure time) are identified depending on the species under cultivation; however, further confirmation testing is necessary before these can be utilised at a large scale. These results will be useful for aquaculturists wishing to maximise tank productivity through the selective removal of an epiphyte and may also be useful for fundamental studies that investigate the physiological mechanism of tolerance seen in specific species. No formulation was successful in selective elimination of epibiotic *Littorina* spp. from *U. lactuca*, although agitation in freshwater maybe sufficient to detach them.
